# Visualization of Keratin with Diffuse Reflectance and Autofluorescence Imaging and Nonlinear Optical Microscopy in a Rare Keratinopathic Ichthyosis

**DOI:** 10.3390/s21041105

**Published:** 2021-02-05

**Authors:** Pálma Anker, Luca Fésűs, Norbert Kiss, Judit Noll, Krisztina Becker, Enikő Kuroli, Balázs Mayer, Szabolcs Bozsányi, Kende Lőrincz, Ilze Lihacova, Alexey Lihachev, Marta Lange, Norbert Wikonkál, Márta Medvecz

**Affiliations:** 1 Department of Dermatology, Venereology and Dermatooncology, Semmelweis University, 1083 Budapest, Hungary; anker.palma@phd.semmelweis.hu (P.A.); luca.fesus@gmail.com (L.F.); norbert.f.kiss@gmail.com (N.K.); becker.krisztina@gmail.com (K.B.); kuroli.eniko@med.semmelweis-univ.hu (E.K.); mayer.balazs@med.semmelweis-univ.hu (B.M.); bozsanyiszabolcs@gmail.com (S.B.); lorinczkende@gmail.com (K.L.); wikonkal@gmail.com (N.W.); 2 Department of Dermatology, Szent Janos Hospital, 1125 Budapest, Hungary; lupusetuxor@gmail.com; 3 1st Department of Pathology and Experimental Cancer Research, Semmelweis University, 1083 Budapest, Hungary; 4 Institute of Atomic Physics and Spectroscopy, University of Latvia, LV-1586 Riga, Latvia; ilze.lihacova@gmail.com (I.L.); aleksejs.lihacovs@gmail.com (A.L.); marta.lange.rtu@gmail.com (M.L.)

**Keywords:** KRT1, keratin, epidermolytic ichthyosis, nonlinear microscopy, multiphoton microscopy, LED, diffuse reflectance, hyperkeratosis, autofluorescence, histopathology

## Abstract

Keratins are one of the main fluorophores of the skin. Keratinization disorders can lead to alterations in the optical properties of the skin. We set out to investigate a rare form of keratinopathic ichthyosis caused by *KRT1* mutation with two different optical imaging methods. We used a newly developed light emitting diode (LED) based device to analyze autofluorescence signal at 405 nm excitation and diffuse reflectance at 526 nm in vivo. Mean autofluorescence intensity of the hyperkeratotic palmar skin was markedly higher in comparison to the healthy control (162.35 vs. 51.14). To further assess the skin status, we examined samples from affected skin areas ex vivo by nonlinear optical microscopy. Two-photon excited fluorescence and second-harmonic generation can visualize epidermal keratin and dermal collagen, respectively. We were able to visualize the structure of the epidermis and other skin changes caused by abnormal keratin formation. Taken together, we were able to show that such imaging modalities are useful for the diagnosis and follow-up of keratinopathic diseases.

## 1. Introduction

Keratins function as the main structural proteins of the keratinocyte cytoskeleton. Keratin 1 and 10 form heterodimers in the suprabasal post-mitotic keratinocytes. For these reasons, mutations of keratin genes lead to skin keratinization disorders. Dominant negative mutations of the genes that encode keratin 1 and 10 (*KRT1*, *KRT10*) cause epidermolytic ichthyosis (EI), previously also known as epidermolytic hyperkeratosis and bullous congenital ichthyosiform erythroderma of Brocq. Palmoplantar keratoderma (PPK) is usually associated with *KRT1* mutations [1]. Mutations of genes that encode keratins, including *KRT1*, are mostly missense mutations that affect the helix initiation (1A) and helix termination (2B) motifs, which are highly conserved regions of approximately 20 amino acids at the beginning and end of the central helical coiled-coil rod domain [2]. However, disease-causing mutations can be scattered throughout the keratin 1 protein to result in complex genotypic and phenotypic variability of keratinopathic ichthyoses [1]. Keratins are also one of the several endogenous fluorophores in the skin in addition to collagen, elastin, and tryptophan [3,4,5,6,7]. A reliable noninvasive way to analyze skin changes in skin diseases with abnormal keratinization would be of interest. Autofluorescence imaging under narrow band light emitting diode (LED) excitation uses narrow spectral light of different wavelengths to determine the distribution of the endogenous fluorophores of the skin. Using 405 nm wavelength illumination, skin autofluorescence occurs that is mainly attributed to keratins [8,9]. Nonlinear microscopy (NLM) is a promising method for label-free imaging, mostly used in brain research. NLM techniques including two-photon absorption fluorescence (TPF) and second-harmonic generation (SHG) can be utilized for the assessment of the skin with high tissue resolution. High-photon intensity is required for the generation of nonlinear optical processes, which can be achieved by femtosecond or picosecond pulse laser systems operating in the near infrared (NIR) spectrum [10]. Moreover, while the excitation is in the NIR wavelength, the emitted photons are in the visible light spectrum. TPF allows the visualization of keratins, elastin, and melanin, whereas SHG can be utilized for the imaging of collagen [11]. Recently our group used these two modalities in the ex vivo assessment of pseudoxanthoma elasticum and Ehlers–Danlos syndrome [11,12]. Here, we examined the skin lesions of a 3-year-old EI patient in vivo with autofluorescence imaging under narrow band LED excitation and ex vivo with NLM with particular focus on the visualization of alterations brought on by hyperkeratosis.

## 2. Materials and Methods

### 2.1. Mutation Analysis

Genomic DNA was isolated from peripheral blood leukocytes of the patient and his parents. Exons and flanking intron regions were amplified using pre-designed *KRT1*-specific primers attached to M13 tail sequences in the VariantSEQr PCR sequencing system (Applied Biosystems, Foster City, CA, USA). The brief description of all the steps of genomic DNA isolation and Sanger sequencing was published earlier [13].

### 2.2. Sample Preparation

Skin biopsies were taken with a 4 mm punch biopsy tool. Two samples were collected from the plantar region of the keratoderma patient for NLM imaging. One fresh skin biopsy specimen was transported in phosphate-buffered saline and placed on slides to obtain z-stack images with NLM. The other biopsy sample was formalin-fixed, paraffin-embedded and 20 μm thick sections were prepared. After deparaffinization, unstained sections were covered with coverslips to acquire vertical NLM images. Hematoxylin and eosin (H&E)-stained sections according to standard methods were used for histopathologic analysis. One identically treated set of samples from an uninvolved individual served as control.

### 2.3. Autofluorescence Imaging under Narrow Band LED Excitation

A newly developed LED device was used, as described previously [8,14]. A set of autofluorescence images under continuous 405 nm LED excitation was recorded. In addition, diffuse reflectance images were acquired under 526 nm illumination. Four battery-powered violet and green LEDs were placed within a cylindrical lightshielding wall that also ensured fixed distance (60 mm) between the camera and the evenly illuminated skin. A long pass filter (>515 nm) was placed in front of a color CMOS 5 megapixel IDS camera (MT9P006STC, IDS uEye UI3581LE-C-HQ, Obersulm, Germany) to prevent detection of 405 nm LED emission. The skin of our patient was assessed at two different sites to compare the endogenous autofluorescence of uninvolved skin with the changes caused by hyperkeratosis. An age- and gender-matched healthy child was assessed in the same manner as control. On all images, affected and uninvolved skin areas were selected manually as regions of interest (ROI). We analyzed the mean AF and mean diffuse reflectance intensity values within the selected ROIs with ImageJ v1.52a software (NIH, Bethesda, MD, USA).

### 2.4. Nonlinear Microscopy Imaging

NLM imaging was performed using a ≈20 MHz repetition rate, sub-ps Ti:Sapphire laser (FemtoRose 300 TUN LC, R&D Ultrafast Lasers Ltd., Budapest, Hungary), and a commercial Axio Examiner LSM 7 MP laser scanning 2P microscope with a 20×, 1.0 NA water immersion objective (W-Plan-APOCHROMAT, Carl Zeiss Microscopy GmbH, Jena, Germany). The central wavelength of the pump laser was set to 800 nm, with a bandwidth of <2 nm. A 405/20 nm bandpass filter was used to collect the SHG signal and a 590/45 nm (orange) bandpass filter was used to collect the TPF signal before the non-descanned detection (NDD) detectors. Two-channel, 16 bit images were captured from individual imaging areas of 420 × 420 μm^2^; the pixel dwell time was set to 12 μs. 2D mosaic images of vertical skin sections were captured. The acquired TPF and SHG images were merged and assembled into two-channel images with ImageJ v1.46 software (NIH, Bethesda, MD, USA). Z-stacks from the skin biopsy samples were obtained by the Zeiss Zen software v3.0 (Carl Zeiss AG, Germany) with 5 μm steps between the horizontal images. 3D images were generated from these 2D images along the *z*-axis with ImageJ software.

## 3. Results

### 3.1. Case Report

**Figure 1:**
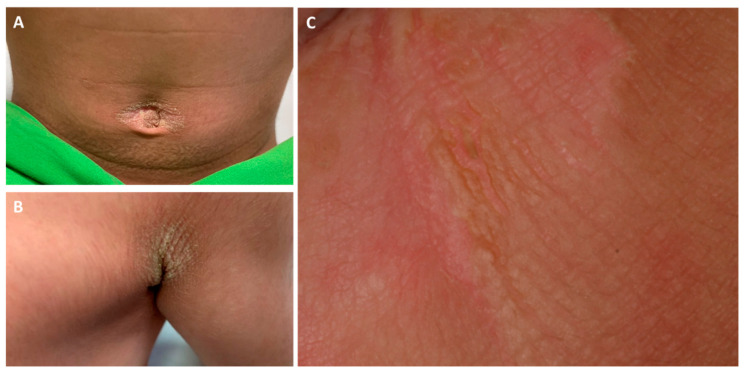
Clinical pictures of the umbilical (**A**) and axillar (**B**) regions of the proband. The gluteal region (**C**) showed a marked hyperkeratotic plaque with mild epidermolysis, fissures and lichenification.

**Figure 2:**
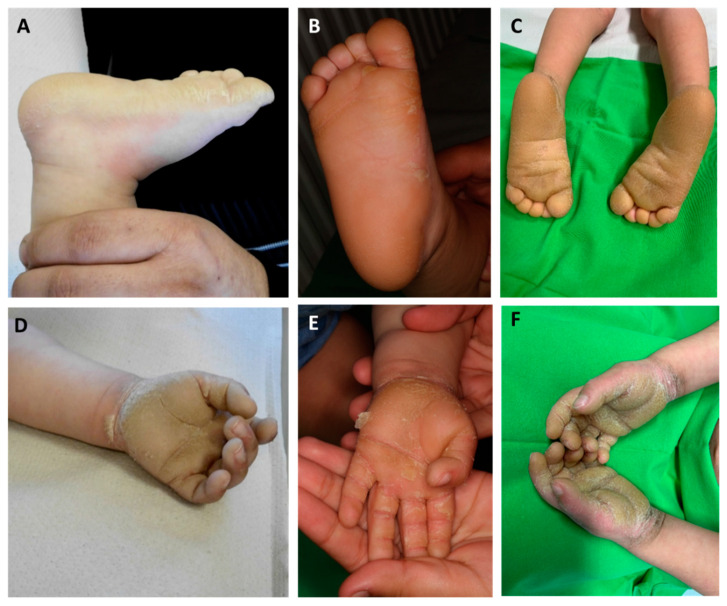
Palmoplantar keratoderma of the patient at the age of 6 months (**A**,**D**), 1 year (**B**,**E**), and 3 years (**C**,**F**). Extension of hyperkeratosis beyond the palmoplantar margins can be observed. In our case, the severity of palmoplantar keratoderma (PPK) showed changes over time.

Here, we report the case of a 3-year-old boy presenting with brownish erythematous scaly plaques in the axillar, popliteal, umbilical, and inguinal regions with diffuse PPK (Figure 1 and Figure 2). At birth, the patient had blisters and erosions, and marked scaling was present on the scalp, flexural and intertriginous areas, palms, and soles. The severity of the symptoms fluctuated over time, occasionally with more severe epidermolysis, fissures, and onychodystrophy. The parents had no relevant history of skin disease.

### 3.2. Mutation Analysis

**Figure 3:**
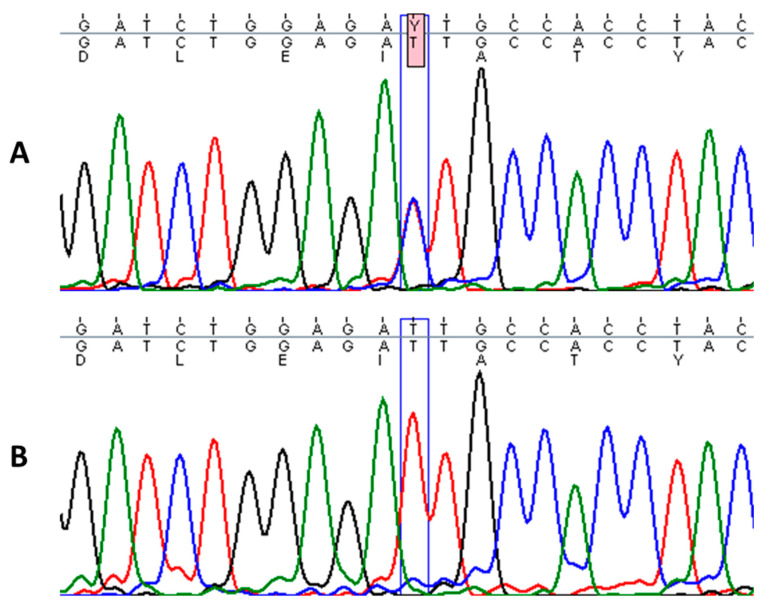
Sequence analysis of the affected patient with heterozygous mutation c.1436T > C in *KRT1* gene (**A**) and homozygous wild-type sequence in a healthy individual (**B**).

Sequencing of *KRT1* gene revealed a heterozygous missense mutation c.1436T > C in the proband, designated as p.Ile479Thr, which leads to a change of isoleucine to threonine in the highly conserved 2B domain of keratin 1 (Figure 3). This particular mutation has been described in a few cases of annular epidermolytic ichthyosis (AEI, OMIM: 607602) and in the background of the major form of EI (OMIM: 113800), resulting in distinct phenotypes [15,16]. Genetic tests from the parents did not identify this pathogenic variant. Additionally, an in-frame, 21-bp deletion variant in exon 9 of *KRT1* (p.Gly553-Tyr559del) was found in the proband and his mother [17].

### 3.3. Autofluorescence Imaging under Narrow Band LED Excitation

**Figure 4:**
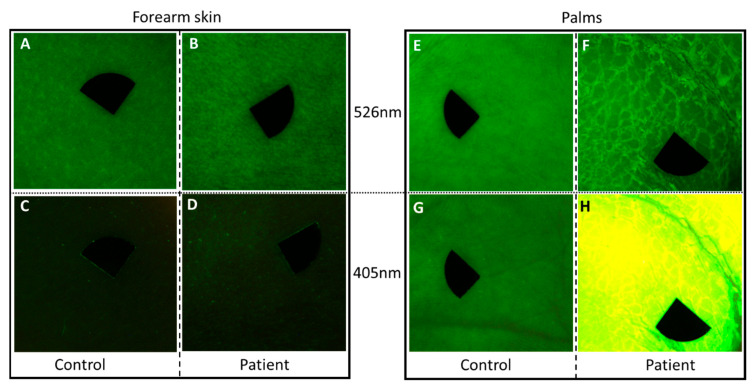
Diffuse reflectance images of the patient and an age-matched healthy individual at 526 nm (**A**,**B**,**E**,**F**) and autofluorescence under 405 nm light emitting diode (LED) excitation (**C**,**D**,**G**,**H**). (**A**–**D**) Clear skin area on the forearm of the patient and the healthy control with the endogenous autofluorescence of the skin; (**E**–**H**) palmar skin; (**F**) the edges of the thick squames were defined at 526 nm illumination on the patient; (**H**) the thick hyperkeratotic layer on the palm gave a strong autofluorescence signal at 405 nm excitation. The size of the images is 2 × 2 cm^2^. Black markers do not point to areas of interest, but are used for image alignment. Pixel size: 180 pixels/cm.

Compared to the uninvolved skin region on the arm of the patient and both assessed skin sites of the healthy control, the thick palmar hyperkeratosis showed marked autofluorescence at 405 nm excitation (Figure 4). Additionally, a lower diffuse reflectance signal could be detected with the 526 nm illumination in this case. Quantitative analyses revealed high mean AF intensity in the palmar hyperkeratosis compared to clear palmar skin and unaffected skin area of the patient (162.35 vs. 51.14 vs. 20.46). The mean AF intensity in case of the unaffected region of the patient and the healthy control showed a slightly lower value (20.46 vs. 35.97). In the case of palmar skin, mean intensity values of the diffuse reflectance images were higher in the patient and control (45.83 and 48.12, respectively) compared to forearm skin of the patient and the control (35.01 and 36.01, respectively). Mean intensity values of diffuse reflectance imaging did not show considerable difference between the patient and the healthy control.

### 3.4. NLM Imaging and Histology

**Figure 5:**
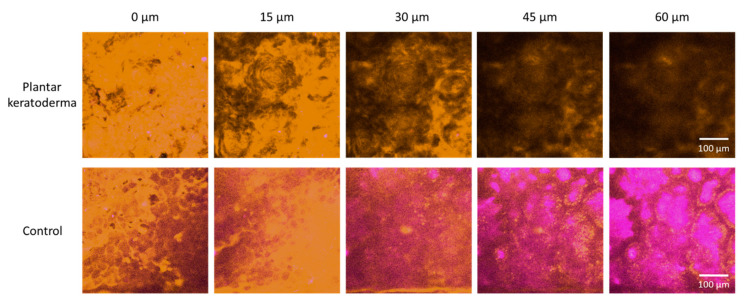
Nonlinear microscopy (NLM) horizontal optical slicing through skin biopsies from the epidermis towards the dermis. Upper panel: plantar keratoderma of the patient lower panel: healthy control. Representative images acquired at five different vertical positions along the *z*-axis, 15 μm apart in each case, through the skin biopsies positioned horizontally on the microscopy slides. Orange color (590/45 nm bandpass emission filter) and magenta color (405/20 nm bandpass filter) indicate two-photon absorption fluorescence (TPF) and second-harmonic generation (SHG) signals, respectively, in the imaging plane. Scale bar: 100 μm. Pixel size: 0.8476 pixels/µm.

**Figure 6:**
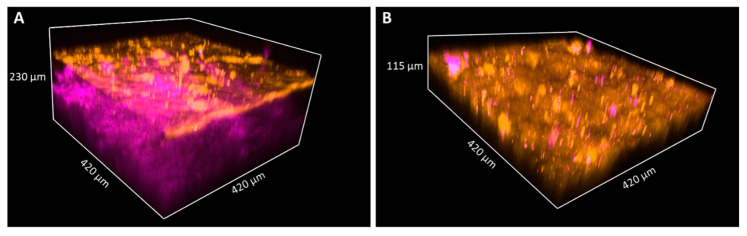
TPF and SHG images of keratin and collagen in 3D images of skin biopsies of healthy subject (**A**) and palmoplantar keratoderma (**B**). Penetration depth of the pump laser markedly decreases if the epidermis expands; thus, the papillary dermis could not be reached in the case of keratoderma. The size of the images as follows: 420 × 420 μm.

**Figure 7:**
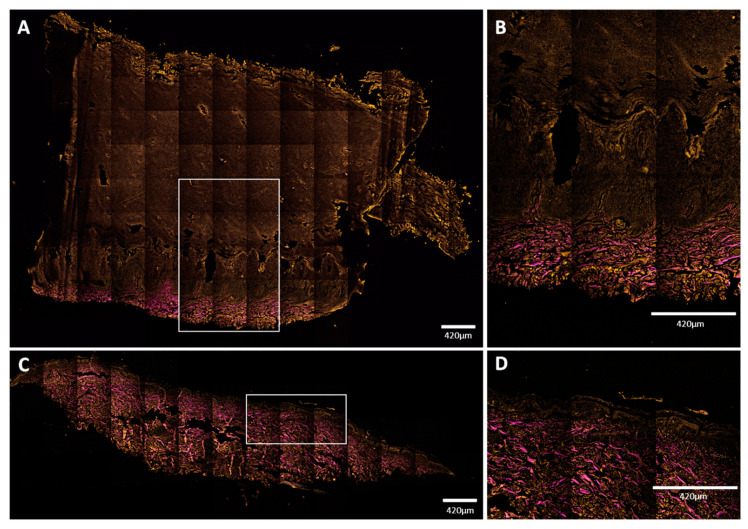
Mosaic NLM images of vertical skin sections. (**A**,**B**) Plantar keratoderma of the patient; (**C**,**D**) healthy control. Keratin had TPF signal in the orange channel, while collagen had SHG signal, indicated with magenta color. (**A**,**B**) Dermal papillae appeared in magenta; upwards, the basal keratinocyte layer can be seen in orange, followed by widened cellular layers of the epidermis and the increased amount of lamellar structure of the cornified keratinocytes. Total thickness of the epidermis was around 3000 μm (range: 3079–3373 μm). (**C**,**D**) The structure and proportion of the epidermis appeared to be as assumed in healthy subjects. Thickness of the epidermis was about 100 μm (range: 96–146 μm). Scale bar: 420 μm. Pixel size: 0.1571 pixels/µm (**A**,**C**); 0.4012 pixels/µm (**B**,**D**).

**Figure 8:**
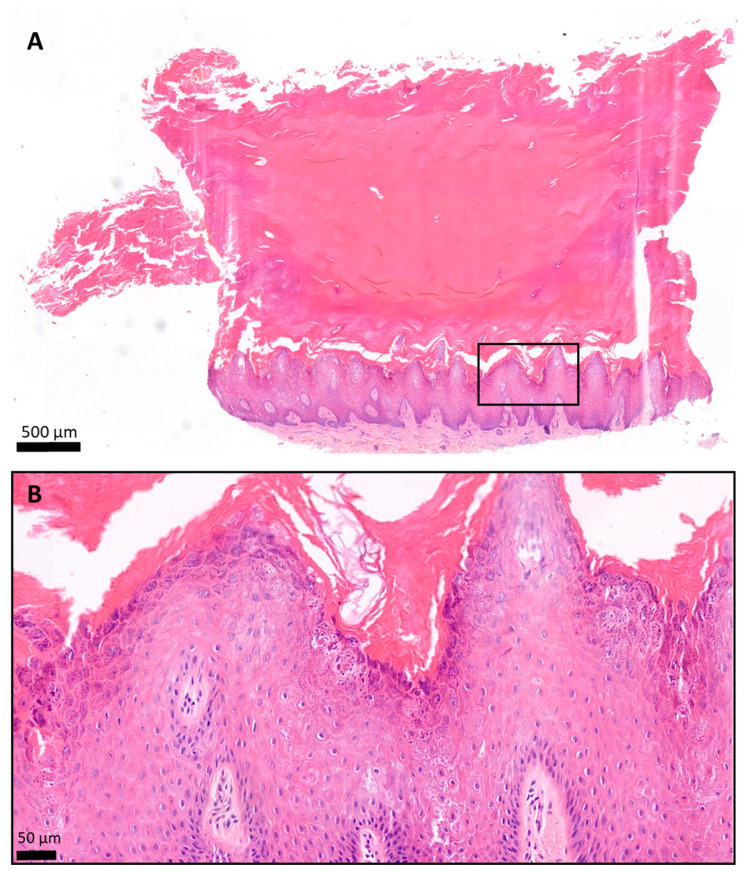
Histology of the plantar keratoderma of the patient, hematoxylin and eosin (H&E) staining. The skin biopsy specimen had a structure typical of acral localization. (**A**) The surface was covered by a very thick hyperkeratotic and orthokeratotic horny layer. (**B**) The epidermis was significantly acanthotic and slightly papillomatous. The stratum granulosum was widened. The keratohyaline granules were differently shaped and sized and showed perinuclear vacuolization of the keratinocytes. The basal membrane was intact. (**A**) 2.8× magnification; scale bar: 500 µm; pixel size: 0.184 pixels/µm. (**B**) 19.9× magnification; scale bar: 50 µm; pixel size: 1.12 pixels/µm.

In the fresh skin biopsy sample, the papillary dermis could not be visualized in the plantar keratoderma sample due to the thick hyperkeratosis and strong keratin autofluorescence that had a considerable scattering effect and thus decreased the depth of penetration (Figure 5 and Figure 6). The maximum depth in the skin was 115 µm and 230 µm in the cases of the plantar keratoderma and the control sample, respectively (Figure 6). Formalin-fixed, previously paraffin-embedded, deparaffinized sections were assessed with TPF and SHG as well, where 2D vertical cross-section mosaic images were acquired to mimic conventional histology. On the bottom of the plantar keratoderma sample, the papillary dermis could be seen by the visualization of collagen via SHG (Figure 7). The cellular part of the epidermis appeared in the TPF channel between the dermal papillae. By moving upward, cellular structures could no longer be identified and were replaced by lamellae in the TPF channel that represented the cornified keratinocytes and protein aggregates. Corresponding histologic changes could be identified in the H&E-stained sections (Figure 8). On the NLM image of the control skin sample, the thin layer of the epidermis and the collagen network of the dermis and subcutis could be visualized.

## 4. Discussion

Ichthyoses are a group of rare genetic skin diseases with a heterogenous genetic background that results in diverse phenotypes of keratinization disorders. Mutations of the suprabasal epidermal cytokeratin genes (*KRT1* and *KRT10*) are responsible for EI, as their protein products account for the integrity of keratinocytes. EI affects approximately 1 in 300,000 individuals. It is known that 50% of EI cases are inherited from an affected parent, while the other half of the cases are due to de novo mutations [18]. The parents of our patient had no history of keratinization disorder, and genetic testing revealed only wild-type alleles in both parents. Thus, this case was likely due to either a spontaneous mutation of the patient or a germline mutation in one of his parents. Here, c.1436T > C mutation was detected by sequencing of *KRT1*, which resulted in an isoleucine-to-threonine amino acid change in the helix termination motif (2B) of keratin 1 [15]. Mutations that affect such conserved regions have been considered to associate with severe EI disease phenotypes since they are important for keratin assembly and filament formation. However, c.1436T > C has been described to cause both severe and mild EI phenotypes including AEI (OMIM: 607602). Although EI subtypes are similar at birth with blistering and erosions, the subsequent symptoms of AEI and major EI types are different, with AEI resulting in a milder phenotype [16]. Our patient did not show characteristics of AEI, namely, recurrent flares of annular erythematous lesions with periods of almost clear skin, yet cutaneous symptoms with relapses and remissions were seen. All reported cases with p.Ile479Thr were associated with severe PPK, regardless of EI subtype. Two families were reported thus far as atypical epidermolytic PPK with the same *KRT1* mutation where cutaneous symptoms were modest besides PPK extending beyond the palmoplantar margins [19]. Hyperkeratosis is limited only to the palmoplantar surfaces in the case of typical PPK associated with *KRT9* mutations. In line with the literature, PPK of our patient progressed over time. Additionally, in these two families of atypical PPK, the same heterozygous 21-bp in-frame deletion was present in exon 9 of *KRT1*, as we observed in our patient and his mother [19]. Gly553-Tyr559del was reported to cause size polymorphism of keratin 1 with a frequency of 39% in the general population [17]. Overall, the genotype–phenotype correlation in PPK in EI is well established, and its presence is associated with *KRT1* but not *KRT10* mutations [20]. However, our case supports the notion that for skin symptoms, such as hyperkeratosis, blistering, erythema, and the severity of the disease, the correlation is loose. The loose genotype–phenotype correlation is hypothesized to be due to yet unidentified genetic and epigenetic factors [16]. Although the diagnosis of ichthyoses is based on genetical testing, conventional histology is often used preceding genetical analysis or in cases when a genetical background cannot be identified. Our aim was to investigate two different optical modalities that could be useful in the diagnosis of disorders of keratinization besides conventional histology. Keratins are ideal targets for imaging methods as they are one of the major fluorophores of the skin besides lipids, collagen, and elastin. Light absorption and emission depends greatly on the fluorophore and chromophore content and distribution of the skin, and thus biological processes that affect these can consequently change the optical properties of the tissue [21]. Alteration of tissue autofluorescence has been of particular interest in skin cancer research and diagnostics [8,9,14,22]. However, a better characterization of the optical properties of skin tissue components by studying genetic disorders could provide valuable data for noninvasive optical diagnosis of skin diseases. Here, we compared the skin lesions of our patient with a healthy control using autofluorescence imaging under narrow band LED illumination. Hyperkeratosis produced high autofluorescence signal under 405 nm LED excitation [23]. Hyperkeratosis by definition is an abundance in keratin, providing a strong autofluorescence signal. Yet, we presume that not all the signal comes from the keratin, since other fluorophores, including lipids, might be involved [24]. Although the exact cause of the strong autofluorescence was not clear, the effect of hyperkeratosis on changes of intrinsic autofluorescence was considerable (Figure 4). AF patterns of skin with variable keratin content were investigated in case of hand callus, palmar skin, and thin forearm skin at different exciting wavelengths. Emission and excitation scans showed significant differences between forearm skin and callus that were mainly attributed to the increased keratin content, whereas normal thickness palmar skin and callus had similarities in their AF pattern [7]. AF changes in cases of inflammatory skin diseases, with psoriasis and atopic dermatitis having been assessed under 408 nm LED excitation and being proved to be a valuable tool in the improvement of diagnostic accuracy. There, the authors attributed the high AF intensity of psoriasis to abnormal keratinization among other factors, such as increased involucrin levels and increased collagen production [25]. The fluorescence signal due to LED excitation is a mixture of signals from different tissue layers. Hence, signals originating from different layers can cause interference [26]. A hyperkeratotic epithelial layer may reduce autofluorescence of underlying tissue by attenuating the penetration of the excitation light [23]. NLM can eliminate this problem by providing optical sectioning, which means the separation of autofluorescence signals from different tissue layers [24]. Additionally, different NLM modalities such as TPF and SHG allow a selective visualization of different tissue components. Thick hyperkeratosis of the plantar keratoderma sample significantly impaired penetration depth. Even though the papillary dermis could be identified on the vertical images of the skin section, it could not be reached with horizontal optical sectioning of the fresh skin biopsy sample due to the scattering effect of keratin. In the case of the healthy control, normal epithelial structure allowed for a clear visualization of this layer. Additionally, this method could be valuable in the diagnosis of keratinization disorders without a hyperkeratotic epidermis. Although vertical imaging with NLM requires biopsy, it provides more detailed image of the corneal layer compared to conventional histology. Thus, it could be valuable in the examination of diseases affecting the stratum corneum. In conclusion, we introduced ex vivo NLM and in vivo autofluorescence imaging under narrow band LED excitation for the visualization of keratin in the case of a rare keratinopathic ichthyosis. NLM provides cell level resolution with optical sectioning. However, hyperkeratosis can hinder NLM imaging in the case of fresh biopsy samples. In addition, due to the high cost of NLM, access to such imaging devices is very limited. Autofluorescence imaging under narrow band LED excitation could be a cost-efficient and easy-to-use in vivo method for diagnostics of keratinization disorders.

## Data Availability

Not applicable.
